# Plant-Derived Compounds and Extracts as Modulators of Plasmin Activity—A Review

**DOI:** 10.3390/molecules28041677

**Published:** 2023-02-09

**Authors:** Joanna Kolodziejczyk-Czepas, Jan Czepas

**Affiliations:** 1 Department of General Biochemistry, Faculty of Biology and Environmental Protection, University of Lodz, 90-236 Lodz, Poland; 2 Department of Oncobiology and Epigenetics, Faculty of Biology and Environmental Protection, University of Lodz, 90-236 Lodz, Poland

**Keywords:** fibrinolysis, modulators, plasmin, peptides, phytochemicals, plant extracts

## Abstract

Functionality of the fibrinolytic system is based on activity of its central enzyme, plasmin, responsible for the removal of fibrin clots. Besides the hemostasis, fibrinolytic proteins are also involved in many other physiological and pathological processes, including immune response, extracellular matrix degradation, cell migration, and tissue remodeling. Both the impaired and enhanced activity of fibrinolytic proteins may result in serious physiological consequences: prothrombotic state or excessive bleeding, respectively. However, current medicine offers very few options for treating fibrinolytic disorders, particularly in the case of plasmin inhibition. Although numerous attempts have been undertaken to identify natural or to develop engineered fibrinolytic system modulators, structural similarities within serine proteases of the hemostatic system and pleiotropic activity of fibrinolytic proteins constitute a serious problem in discovering anti- or profibrinolytic agents that could precisely affect the target molecules and reduce the risk of side effects. Therefore, this review aims to present a current knowledge of various classes of natural inhibitors and stimulators of the fibrinolytic system being well-defined low-molecular plant secondary metabolites or constituents of plant extracts as well as plant peptides. This work also discusses obstacles caused by low specificity of most of natural compounds and, hence, outlines recent trends in studies aimed at finding more efficient modulators of plasmin activity, including investigation of modifications of natural pharmacophore templates.

## 1. Introduction

**Figure 1:**
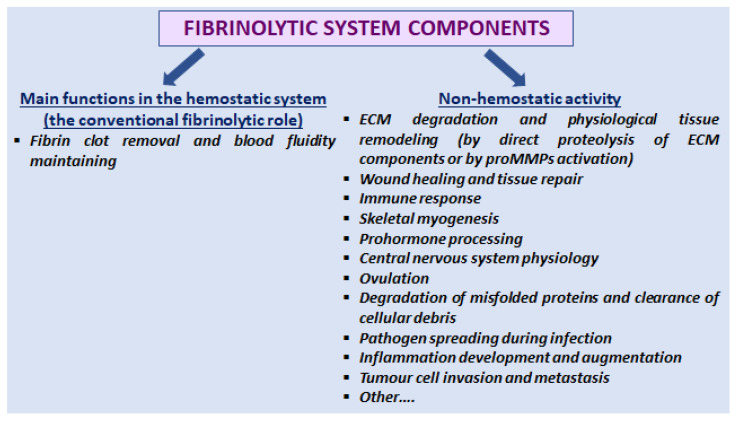
Brief representation of hemostatic and non-hemostatic activity of the fibrinolytic system. ECM—extracellular matrix; proMMPs—matrix metalloproteinase zymogenes.

Among complex mechanisms of hemostasis, the fibrinolytic system has been primarily considered a natural counterbalance of the blood coagulation cascade, responsible for degradation of fibrin clots and maintaining blood fluidity. Physiology of the fibrinolytic system is based on proteolytic activity of the plasmin enzyme and different regulatory mechanisms preserving the hemostatic balance and preventing an excessive proteolysis within blood vessels and tissues surrounding the vasculature. However, removal of fibrin clots is only a part of pleiotropic activity, both of plasmin itself and other fibrinolytic proteins. Functions of the fibrinolytic system extend beyond blood physiology and vascular hemostasis and include participation in extracellular matrix (ECM) degradation, tissue remodeling, wound healing, immune response, and other physiological and pathological processes [1,2,3] (Figure 1). Besides degradation of fibrinogen and fibrin, plasmin hydrolyzes many other protein substrates, including blood coagulation factors (V, VIII, IX, X, von Willebrand factor, FXII, and pre-kallikrein), vascular endothelial growth factor, C3 and C5 complement components, protease-activated receptor-1 (PAR-1), matrix metalloproteinase zymogenes (proMMPs), as well as ECM proteins, e.g., fibronectin, tenascin, collagen, and laminin [4].

Due to an increased risk of thrombo-embolic complications in diseases of civilization, including atherosclerosis, metabolic syndrome, obesity, and cancer, numerous studies have been undertaken to improve diagnostics of coagulation cascade abnormalities and current anticoagulant therapies, as well as to develop novel therapeutic strategies. Many of the aforementioned studies have been mostly focused on inhibition of key proteinases of the blood coagulation cascade and reduction in platelet activity [5,6,7,8,9]. This imbalance between the blood coagulation-centered approach and fibrinolysis-targeting studies has partly contributed to less advanced development of fibrinolysis-targeting therapy. Current medicine offers only three inhibitors to reduce fibrinolysis and few activators of fibrinolysis [10].

Although plasminogen deficiency is very rare (1.6 per million) [11], diverse types of fibrinolysis disorders occur in many other diseases. Under pathological conditions associated with a pro-coagulant state, impaired fibrinolysis becomes an additional risk factor of thrombosis [12,13]. On the other hand, an increased activity of the fibrinolytic system is a risk of uncontrolled bleedings associated with different surgical procedures [14,15]. Antifibrinolytics are used in patients with von Willebrand disease [16] and to treat thrombolytic-induced bleedings [17]. Although antifibrinolytics may be helpful in the treatment of mucosal bleedings, they should be administered with caution to patients with congenital fibrinogen disorders with a personal or family history of thrombotic episode [18].

Fibrinolysis is a tightly regulated process, involving interactions of the fibrin surface, plasminogen, and its main activator, i.e., the tissue-type plasminogen activator (t-PA). It begins with formation of the fibrin clot and the activation of t-PA. Release of t-PA from endothelial cells is a critical step in intravascular fibrinolysis, enabling the conversion of the plasminogen zymogen into the plasmin enzyme [19]. Fibrinogen mediates both the coagulation and fibrinolysis processes through thrombin binding and limiting its activity as well as through interactions with the coagulation factor FXIII, plasminogen, t-PA, and antiplasmin [20]. Preliminary efforts to recognize possibility of fibrinolysis modulation by natural compounds or extracts originated from plants were undertaken as early as the 1970s and 1980s [21]. From that time, many interesting results have appeared. This review aims to summarize a current state of the art in the field of plant-derived modulators of fibrinolysis, in the context of a search for inhibitors of fibrinolytic proteins and substances displaying thrombolytic or fibrinolysis-stimulatory properties.

## 2. Plasminogen Structure and Main Components of the Fibrinolytic System

**Figure 2:**
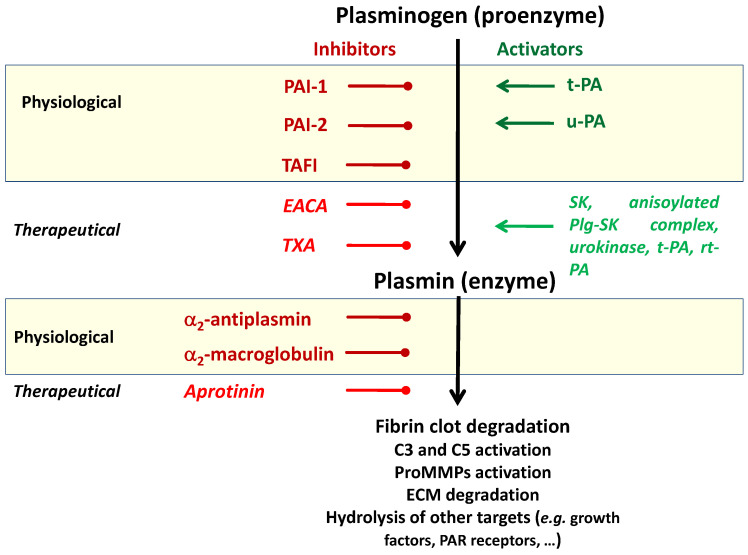
Main components of the fibrinolytic system and their physiological and therapeutical activators and inhibitors. C3, C5—complement components; EACA—aminocaproic acid; ECM—extracellular matrix; PAI-1, PAI-2—plasminogen activator inhibitors 1 and 2; PAR—protease-activated receptor; Plg-SK—plasminogen-streptokinase; proMMPs—matrix metalloproteinase zymogenes; rt-PA—recombinant t-PA; SK—streptokinase; TAFI—thrombin-activatable fibrinolysis inhibitor; t-PA—tissue-type plasminogen activator; TXA—tranexamic acid; u-PA—urokinase-type plasminogen activator.

The functional core of the fibrinolytic system is the plasminogen (zymogene)-plasmin (the active enzyme) axis, controlled by activators and inhibitors (Figure 2). The structure of native plasminogen, named the Glu-plasminogen (Glu-Plg, 92 kDa), comprises 791 amino acids (aa) and is organized into seven domains, as follows: N-terminal Pan-apple (PAp) domain, five homologous kringle domains (K1-K5), and a serine protease (SP) domain. Plasminogen (Plg) is mainly synthesized in the liver and circulates in blood flow at a concentration of about 2 μM (0.18 mg/mL) in two single-chain glycoforms, i.e., glycoform I and II. Hydrolytic removal of the N-terminal peptide (77 aa) from the Glu-Plg molecule by plasmin leads to the formation of a slightly shorter form of the zymogene, i.e., Lys-Plg, which is more prone to the proteolytic activation by plasmin, compared to the Glu-Plg. The kringle domains in the plasminogen/plasmin molecule (about 80 aa) are triple-looped structures, stabilized by three intrachain disulfide bonds and contain the lysine binding sites (LBS). Among five kringle domains, four contain the functional LBS. In the K1, K2, K4, and K5 domains, DXD/E motifs enabling their interaction with lysine ligands are present. The K3 domain contains a DXK sequence instead, which results in the lack of ability to bind lysine. This dysfunctional LBS can be activated as a result of substitution of Lys311 to Asp [22,23,24]. A general scheme of kringle sequences is based on anionic and cationic centers separated by a hydrophobic region. While the anionic centers of K1, K2, K4, and K5 are very similar (including two acidic residues at identical positions), the cationic centers display higher variability. The cationic center of K1 contains two positively charged amino acid residues (i.e., R117 and R153), with a hydrophobic residue of Y146. The K2 cationic center contains only R234. In the cationic center of the K4 domain, K392 and R426 are present. The K5 domain lacks the cationic center [25]. Structural differences between the kringles (and LBS) influence their ligand binding capacity. The K1 and K4 domains were found to display a preference for lysine-like dipolar molecules, and K2 and K5 have a marginal affinity for these types of ligands [26].

In blood flow, plasminogen circulates in a closed conformation, maintained by various intramolecular interactions (for details see: [4,22]). The critical step in plasminogen activation is its LBS-mediated interaction with the target protein or receptors on the cell surface. This interaction initiates conformational changes in the plasminogen molecule, converting its closed structure into open conformation to expose the activation loop. During the immobilization, K2, K4, and K5 domains (bound to the self-lysines/arginines in the closed conformation of plasminogen) are switched onto lysine residues of the target surface. The LBS are responsible for interactions both with fibrinogen/fibrin and targets such as α2-antiplasmin, plasminogen receptors on the cell surface, as well as other ligands [22,23,24,27].

The activation of plasminogen to plasmin occurs through a cleavage of the Arg561-Val562 peptide bond. The plasmin (EC 3.4.21.7) is a two-chain enzyme with an active center (Ser741, His603, and Asp646) located in the B (light) chain (25 kDa). The heavy A chain (60 kDa) contains the kringle structures. Plasminogen conversion to plasmin is primarily mediated by the tissue-type plasminogen activator (t-PA, EC 3.4.21.68) and the urokinase-type plasminogen activator (u-PA/urokinase, EC 3.4.21.73). The t-PA-mediated plasminogen conversion to plasmin is the main mechanism of thrombolysis activation in blood vessels, whereas the u-PA and its receptor are mainly involved in the extravascular proteolysis, including the tissue remodeling. The plasminogen proenzyme can be also proteolytically activated by small amounts of already generated plasmin as well as by interactions with other proteases, including the blood coagulation cascade enzymes such as factors XIa and XIIa and kallikrein. Plasmin is able to amplify fibrinolysis efficiency by a direct activation of plasminogen molecules or by proteolysis of the single chain t-PA and u-PA to their more active, double-chain forms [28].

During the phase of plasminogen conversion to plasmin, activity of the fibrinolytic system is regulated by the plasminogen activator inhibitors (PAIs), members of the SERPINs family. The plasminogen activator inhibitor-1 (PAI-1), able to inhibit both t-PA and u-PA, is the main inhibitor of plasmin generation. The plasminogen activator inhibitor-2 (PAI-2) selectively inactivates u-PA and is considered an additional regulator of plasmin formation. Plasmin activity is inhibited via the formation of irreversible complexes with α2-antiplasmin (the main plasmin inhibitor) and, to a minor extent, by α2-macroglobulin [29]. Indirectly, plasminogen activation and plasmin generation are controlled by the thrombin-activatable fibrinolysis inhibitor (TAFI). This regulator of the fibrinolytic rate removes C-terminal lysine residues from the partially degraded fibrin clot, and therefore it limits plasminogen binding and activation [30].

Biochemical characteristics of main components of the fibrinolytic system are briefly presented in Table 1.

## 3. Current Pro- and Anti-Fibrinolytic Therapies

**Figure 3:**
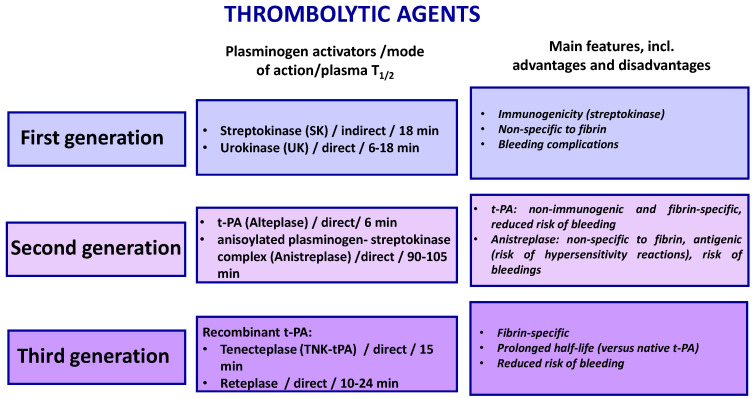
Different generations of thrombolytic drugs.

The number of pharmacological regimens available for patients requiring thrombolytic or antifibrinolytic therapy is limited [31,32,33,34] (Table 2). Currently used drugs enable modulation of the fibrinolytic system activity by regulation (activation or inhibition) of plasminogen conversion to plasmin or by inhibition of plasmin activity. Maintaining the fibrinolytic potential of blood plasma is an important element of anti-thrombotic therapy. Thrombolytic therapy is crucial for dissolving intravascular clots and thus for maintaining blood flow and preventing ischemic damage in patients with acute myocardial infarction, acute ischemic stroke, deep vein thrombosis, pulmonary embolism, and other types of thrombo-embolic complications. It is based on non-fibrin-specific and fibrin-specific plasminogen activators, including exogenous activators such as streptokinase and variants of t-PA, i.e., tenecteplase (TNK-tPA), and reteplase (Figure 3). Adverse effects of this type of treatment are bleeding, hypotension, and allergic reactions (most frequently, after use of streptokinase) [35,36,37].

Possibility of extensive postoperative bleeding or other risks of blood loss are reduced by the antifibrinolytic treatment. At the level of plasminogen activation, fibrinolysis is inhibited by administration of lysine analogues, impairing plasminogen activation to plasmin [38,39,40]. Clinically used drugs belonging to this group (i.e., tranexamic acid (TXA) and aminocaproic acid (EACA)) are capable of blocking the LBS of the kringle domains in the plasminogen molecule, which are crucial for interactions of this protein with fibrin and other target surfaces. The amino group of TXA or EACA is bound to the DXD motif of the LBS anionic centers, and the carboxyl group interacts with the cationic centers [26]. Among four kringle domains containing functional LBS, the K1 displays the highest affinity for both TXA and EACA, followed by K4, K5, and K2 [22,41].

The only currently approved direct inhibitor of plasmin is aprotinin (Trasylol), belonging to the Kunitz-type inhibitors of serine proteases. However, its use in clinical routine has been widely discussed due to adverse effects, including an increased risk of anaphylaxis, renal failure, and mortality. Based on an increasing number of data on serious adverse effects, aprotinin was withdrawn from the European market in 2008. Later, however, clinical use of aprotinin was reacknowledged, but its administration requires careful risk–benefit assessment [42].

## 4. Plant-Derived Inhibitors of the Fibrinolytic System

### 4.1. General Insight into Phytochemicals-Plasmin(ogen) Interactions and the Problem of Selectivity of Compounds

Analogously to physiological mechanisms of fibrinolytic activity control, the possibility of fibrinolysis modulation by plant-derived substances at different molecular levels can also be taken into consideration. Existing reports provide data from tests of different substances, both synthetic and natural, with anti-plasmin activity and emphasize the potential of small molecules. The trypsin-like proteases, including enzymes of the hemostatic system, seem to be prone to action of plant-derived substances, based on different carbon backbones and representing diverse classes of low-molecular phytochemicals, e.g., flavonoids, alcoholic β-glucosides, coumarin derivatives, anthrones, and dianthrones and even the steroid-type compounds such as bufadienolides [43,44,45,46,47,48,49,50,51,52,53,54,55] (Table 3). However, promising outcomes obtained from studies on native or modified peptides encourage further research on plant polypeptides as well. Studies on peptide-based plant inhibitors of serine proteases evidently indicate their higher efficiency when compared to (poly)phenolic compounds, for example, AVPI-12, a protease inhibitor from *Aloe vera* L. leaves with a unique RDWAEPNDGY motif starting its N-terminal region inhibited the plasmin-mediated hydrolysis of human fibrin(ogen) in vitro with efficiency comparable to α2-macroglobulin [56]. Both the Kunitz and other types of peptide inhibitors of different plant origin act mostly at nanomolar and even sub-nanomolar concentrations [57,58,59,60,61,62,63,64,65,66,67,68] (Table 4). In contrast, the most active plant polyphenolics are able to inhibit serine proteases or other enzymes at micromolar concentrations (Table 3).

The central enzyme of fibrinolysis, plasmin, is an obvious target in the search for new regulators of the activity of the fibrinolytic system. However, as a member of the trypsin-like protease group (family S1 (trypsin-like fold), the PA clan), plasmin shares many structural and functional features with other serine proteases, including two β-barrel-like regions in the catalytic domain (described as the “Greek key”), the catalytic triad (composed of His, Asp and Ser) and the nucleophilic attack of the hydroxyl group of the active serine on the carbonyl of the peptide substrate and preferential cleaving on the arginine or lysine [69]. Similarities of the trypsin-like enzymes significantly hinder identification of natural compounds or development of new drugs capable of precisely targeting the activity of plasmin. Even aprotinin, a clinically approved Kunitz-type inhibitor of serine proteases, may interact not only with the active center of plasmin but also with other enzymes, including trypsin, kallikrein, urokinase, elastase, and thrombin. Moreover, the inhibitory efficiency of aprotinin towards plasmin is markedly lower than its ability to block the trypsin enzyme (K_i_ of 0.01 and 0.00006 nM for plasmin and trypsin, respectively) [70]. Therefore, development of an effective and specific plasmin inhibitor formulation is a demanding challenge, but the plant-derived substances are considered as promising pharmacophores, also in the context of selectivity. One previous study screened 55 synthetic, sulfated glycosaminoglycan mimetics, based on nine distinct scaffolds. Among the examined chalcones, flavonoids, sucrose octasulfate, quinazolinones, tetrahydroisoquinolines, flavonoid-quinazolinone heterodimers, bis-quinazolinone homodimers, and bis-flavonoid homodimers, a pentasulfated flavonoid-quinazolinone dimer was the most effective inhibitor of plasmin (IC_50_ = 45 μM). Furthermore, the flavonoid-quinazolinone dimer was selective for plasmin over thrombin and the coagulation factor Xa [71].

### 4.2. Plant-Derived Polypeptide Inhibitors of Fibrinolysis

Plants may be a source of a wide range of peptide-based protease inhibitors such as SERPINs, Kunitz-type inhibitors, Bowman-Birk inhibitors, Kazal-domain inhibitors, cyclic cysteine-rich peptides, phytocystatins, metallocarboxypeptidase inhibitors, potato-type inhibitors, and mustard-type trypsin inhibitors [72,73]. Many of the identified serine protease inhibitors have been isolated from the Leguminosae (Fabaceae) family. The best-known is, of course, the soybean trypsin inhibitor (STI), isolated and characterized by Kunitz [74,75], but plants synthesize a variety of Kunitz-type inhibitors with significant diversity in cysteine content, arrangement of disulfide bonds, as well as other structural and functional differences [76]. For example, *Bauhinia rufa* (Bong.) Steud. seeds contain BrTI, a Kunitz proteinase inhibitor containing the RGD sequence [77], which could be useful in modulation of biological processes which are unrelated to removal of the fibrin clot. The RGD sequence has been found in many integrin ligands (e.g., fibronectin, vitronectin, and fibrinogen) and acts as one of the most important cell adhesion motifs, crucial for the ECM-cell interactions, platelet aggregation, and other protein-cell membrane interactions [78,79]. Incorporation of these three amino acid motifs is considered an important modification in development of new biomaterials and drugs. In the past few years, a formula of cyclic RGD-functionalized liposomes with encapsulated urokinase was proposed for enhancement of thrombolytic efficacy [80].

However, low selectivity still remains one of the major bottlenecks to overcome. While modulation of the hemostatic system requires a precise impact on the target enzymes to recover the fibrinolysis-coagulation balance, plant-derived inhibitors are mostly capable of interacting with both the fibrinolytic enzymes and the coagulation cascade factors (Table 4). Moreover, their inhibitory efficiency towards the coagulation cascade and fibrinolytic proteins may be similar. Although the *Bauhinia* genus has been considered a rich source of natural inhibitors of cysteine and serine proteinases, so far, inhibitors isolated from these plants have been shown to influence the activities of plasmin and coagulation factors with comparable efficiency. For BuXI, one of the *Bauhinia*-derived inhibitors, the plasmin Ki value was established to be 76 nM, and the factor XIIa Ki attained 74 nM [61]. Another Kunitz-type inhibitor, CeKI isolated from *Caesalpinia echinata* Lam., was found to inhibit plasmin and the coagulation factor XIIa activities with the same efficiency, i.e., with an Ki value of 0.18 nM [62]. Some selectivity has been observed for AaTI, a serine protease inhibitor isolated from *Araucaria angustifolia* (Bertol.) Kuntze seeds. The inhibitor did not influence activities of chymotrypsin, human plasma kallikrein, or other coagulation enzymes; however, AaTI was more active towards trypsin (K_i_ = 85 nM) than plasmin (K_i_ = 7.0 μM) [59].

Among plant-derived peptide inhibitors, a 14-amino acid backbone-cyclic sunflower trypsin inhibitor-1 (SFTI-1) seems to be particularly promising. It is the smallest known Bowman-Birk peptide inhibitor, and its structure is stabilized by a single disulfide bond and an intramolecular hydrogen bond network formed between the antiparallel β-strands. The SFTI-1 template, modified by amino acid sequences derived from serpin reactive center loops, has been used to develop selective inhibitors of kallikrein-related peptidases 5 and 7 [81]. The SFTI-1 scaffold was also used as a base for development of a new and effective plasmin inhibitor, called SFTIv3 [82]. Unlike the aprotinin, the designed inhibitor displayed a higher potency towards plasmin (K_i_ = 0.051 nM) than trypsin (K_i_ = 160 nM) and a million-fold selectivity over the S1 family of serine proteases of blood. The inhibitor was characterized by no detectable inhibition of thrombin, FIXa, FXa, FXIa, FXIIa, t-PA, u-PA, plasma kallikrein, or matriptase. Moreover, further studies resulted in development of its rapid synthesis method, based on transient expression of the peptide in *Nicotiana benthamiana* Domin [83].

### 4.3. (Poly)Phenolic Compounds and Other Secondary Metabolites as Inhibitors of Fibrinolysis

Many aspects of plasmin structure and specificity are strictly related to its interactions with (poly)peptide substrates (e.g., preference for Lys over Arg at the P1 position, a slight preference for Tyr over Phe or Trp at the P2 site, and more restricted preferences at other subsites, with reduced levels of lysis of sequences with Trp at the P2 site when Arg was located at the P1 site, were found [84]). These features provide new opportunities for further research and may be useful in development of peptidomimetics of various types (reviewed in [85]) but cannot be directly transferred into the polyphenolic compounds-plasmin interactions. On the other hand, these findings may be helpful in the design of new, combined inhibitors. It has been emphasized that some structural features of both plant-derived and synthetic substances may be essential for their inhibitory action on serine proteases. Three-dimensional QSAR comparative molecular field analysis (CoMFA), employing u-PA, t-PA, plasmin, the coagulation factor Xa, thrombin, and trypsin, suggested the selectivity of molecules with indole/benzoimidazole-5-carboxamidine component towards serine proteases [86]. Some structural similarities of these substances with the quercetin molecule, such as the sterically favorable presence of a phenyl ring and electrostatically interacting hydroxyl group, have been also described [53].

Phytochemicals with two adjacent phenolic hydroxyl groups (or catechol moiety) may be inhibitors of various trypsin-like proteases due to highly conserved structural analogies in enzymes from the S1 family. The X-ray crystallography analyses of the quercetin-u-PA complex structure demonstrated that this flavonoid interacted with the active site of u-PA. The phenyl ring bearing a catechol group (commonly referred also as the B ring) of quercetin was found to insert into the specific substrate binding pocket (S1 pocket), and two hydroxyl groups of the benzene ring (A ring) of the benzopyran part of quercetin molecule interacted with the S2 pocket of u-PA [87]. The role of hydroxyl groups was also confirmed in study on anthocyanidins, which demonstrated the ability of delphinidin, cyanidin, and petunidin to inhibit the u-PA-dependent activation of plasminogen to plasmin. Delphinidin with three hydroxyl groups in the B-ring was the most potent inhibitor, followed by compounds containing two hydroxyl groups in the B-ring, i.e., cyanidin and petunidin. Furthermore, analyses of inhibitory effects of anthocyanidins on the migration of glioblastoma cells involving plasmin suggested an important role of a free hydroxyl group at position 3. Delphinidin was the most effective inhibitor of cell migration, but the presence of sugar residue in position 3 (delphinidin 3-glucoside) abolished the inhibitory action [88]. Recently, pharmacophore modeling, in silico docking, and molecular dynamics simulation of the flavonoid-enzyme interactions demonstrated four features (signed as F1-F4) that may be essential for selective modulation of urokinase. These critical features included the presence of aromatic rings (F1 and F2), connected to Cys191, Gln192, Ser195, and Trp215 residues; a cationic H-bond donor (F3) corresponding to Asp189, Ser190, and Gly219; and a metal ligator (F4) corresponding to Gly216. Based on above features, isorhamnetin, rhamnetin, quercetin, and kaempferol were indicated as the most effective ligands for u-PA [89].

Experiments on plasmin-inhibitory effects of flavonoids isolated from *Blumea balsamifera* DC. demonstrated that two the most effective compounds (IC_50_ = 1.5 and 2.3 µM) had hydroxyl groups at 3′ and 4′ positions. These most efficient inhibitors were characterized by the presence of hydroxyl or methoxyl groups in position 3 and 7, and their efficiency was comparable to leupeptin (IC_50_ = 1.2 µM), used as a reference inhibitor of serine proteases [90]. However, studies on the complement cascade, the activation of which is partly associated with plasmin activity, revealed no inhibition for epicatechin, myricitrin, myricetin, or quercetin, with only weak inhibitory effects of afzelin, quercitrin, kaempferol, and tiliroside (IC_50_ values of 258, 440, 730, and 101 µM, respectively). Anti-complement efficiency of the tested compounds increased in inverse proportion to a number of free hydroxyl groups in the B-ring of a flavonoid structure [91].

### 4.4. Plasmin-Targeting Low-Molecular Plant Metabolites and Their Derivatives

Among natural, non-modified phytochemicals, quercetin is the best-described compound with the serine protease-inhibitory properties. Interactions between this flavonoid and plasmin active center have been visualized in silico by Cuccioloni et al. [92]. Quercetin has been reported to inhibit plasmin, urokinase, thrombin, trypsin, and elastase. Part of the available data indicated competitive characteristics of plasmin inhibition by quercetin and its glycoside derivative, rutin. Molecular docking revealed interactions between the catalytic triad of plasmin (Asp646, His603, Ser741) and quercetin and rutoside (rutin). Other amino acid residues participating in these interactions were Glu606, Arg644, Trp731 for the quercetin- and the Arg644 residue for the rutin-induced inhibition, respectively. Based on the equilibrium dissociation constants, over 10-fold higher affinity of quercetin to plasmin when compared to rutin has been established. The dissociation constants (K_d_) for plasmin-quercetin and plasmin-rutin complexes were 0.715 ± 0.096 and 12.98 ± 0.76 μM, respectively [86]. However, a competitive action of quercetin seems to be one of the possible mechanisms of interaction of this compound with plasmin, and the final effect may be related to the experimental settings, including the used substrate. Comparative studies on inhibitory effects of the plant-derived bufadienolides and quercetin revealed the uncompetitive characteristics of bufadienolide-triggered plasmin inhibition, while the quercetin acted as a non-competitive inhibitor of this enzyme. In silico analyses revealed that quercetin was bound in proximity but not exactly in the active site, independently of the substrate presence or absence in the active site [43]. The aforementioned differences in mechanisms of plasmin inhibition displayed by quercetin, revealed by various research groups, may indicate a mixed type of plasmin inhibition by quercetin as well. Interestingly, different mechanisms of the inhibitory actions were also observed for TXA, a lysine analogue, which typically blocks the LBS. However, under certain experimental conditions, i.e., its high concentrations (∼25 mM), TXA was able to directly inhibit plasmin through binding to the primary S1 pocket of this protease [93]. Diverse effects on plasmin were also found in studies on the bufadienolide rich-fraction isolated from *Kalanchoe daigremontiana* Raym.-Hamet & H. Perrier. This fraction was capable of stimulating or inhibiting plasmin activity, dependently on the applied concentration. While at lower concentrations (i.e., 0.05–2.5 μg/mL) a significant profibrinolytic effect was found, at a higher concentration (i.e., 50 μg/mL), the bufadienolide fraction inhibited the activity of plasmin [43].

The hemostatic-modulatory properties, including inhibition of platelets, changes in erythrocyte membrane fluidity, as well as stimulation or inhibition of coagulation cascade and fibrinolytic proteins, have been also demonstrated for tannins [94]. Browplasminin, a condensed tannin based on heteroflavan-3-ols of catechin with B-type linkages, isolated from flowers of *Brownea grandiceps* Jacq., was identified as an inhibitor of plasmin (IC_50_ = 47.80 μg/mL, K_i_ = 0.76 μg/mL). This tannin did not influence the activity of t-PA or u-PA but slightly inhibited FXa (IC_50_ = 237.08 μg/mL, K_i_ = 61.61 μg/mL) [95]. Animal studies demonstrated diverse effects of tannins on the physiology of the fibrinolytic system. Oral administration of the extract from *Potentilla erecta* (L.) Raeusch. rhizome increased the activity of t-PA (200 mg/kg, for 14 days) in streptozotocin-induced diabetic rats. However, the euglobulin clot lysis time was prolonged [96]. In normoglycemic rats, administration of the extract (400 mg/kg, for 14 days) decreased the activity of t-PA with no effects on PAI-1 [97]. Corilagin, an ellagitannin isolated from *Phyllanthus urinaria* L. aerial parts increased activity of t-PA and reduced the activity of PAI-1 in blood plasma of healthy rats [98].

### 4.5. Natural and Modified Plant Based Inhibitors of Other Components of the Fibrinolytic System

An excessive inhibition of plasmin, a downstream factor involved in multiple critical biological processes, may result in serious physiological consequences, similar to those observed in plasminogen deficiency. Plasminogen-deficient animals have been shown to suffer from growth disorders, impaired fertility, and low survival [99]. For that reason, the upstream factors such as uPAR, u-PA, t-PA, and PAI-1 have been suggested as more specific and safer targets in the context of clinical treatment [100]. Accordingly, both natural (tannic acid (TA), epigallocatechin monogallate (EGCG), epigallocatechin-3,5-digallate (EGCDG), hexachlorophene, quinalizarin, gallic acid, and sennoside A) and synthetic polyphenolic inhibitors have been investigated. Sennoside A, representing dianthrone compounds, was the weakest inhibitor of PAI-1 activity (IC_50_ = 51.0 µM). TA, EGCG, EGCDG, and gallic acid backbones contain galloyl or gallo-galloyl moieties. However, their inhibitory efficiency towards PAI-1 was only partly related to the number of these galloyl units, suggesting that the orientation of units and other factors are also important. While a monomeric compound, i.e., gallic acid (IC_50_ = 6.6 µM), was 1000-fold less active compared to oligomeric TA (IC_50_ = 0.007 µM), EGCG (IC_50_ = 0.091 µM) was only about 10-fold weaker than TA [101].

The u-PA-inhibitory effects were found for crude methanol extracts from six medicinal plant species, i.e., *Croton lucidus* Gage (the most effective one, IC_50_ = 13.52 µg/mL), *Erythroxylum aerolatum* L., *Tabebuia heterophylla* Britton, *Lantana camara* L., *Cananga odorata* (Lam.) Hook.f. & Thomson, and *Amyris elemifera* Kuntze. The chloroform fraction from *C. lucidus* leaves was the most effective u-PA inhibitor (IC_50_ = 3.52 µg/mL) [102]. Quercetin was reported to inhibit the activity of u-PA with an IC_50_ of 12.1 [50] or 20 μM [49]. This plasminogen activator was also inhibited by esculin, rutin [46], silybin, hypericin, and hyperoside [50]. Furthermore, available studies indicate a possibility of urokinase and its receptor control by plant substances at the level of protein expression, especially in the context of anti-cancer therapy [103,104] and modulation of other processes associated with cell migration and ECM degradation [105].

It has been also suggested that selective modifications of polyphenols, such as lipophilization with fatty acids, may represent a new approach in development of inhibitors of serine proteases, including the urokinase. Screening of the protease-inhibitory efficiency of rutin, phloridzin, and esculin esters revealed that the most potent serine protease inhibitors were rutin derivatives, containing medium to long as well as mono- and polyunsaturated fatty acids. However, in the case of inhibition of urokinase, none of the examined esters was more efficient than rutin. The established IC_50_ values for rutin and its most efficient derivatives, i.e., rutin butyrate and rutin arachidonate, were as follows: 6, 8, and 9 μM, respectively [49].

## 5. Pro-Fibrinolytic Effects of Natural, Plant-Derived Substances

Numerous reports devoted to pro-fibrinolytic or thrombolytic activity of different plant extracts or isolated compounds have been published so far [106,107,108,109,110,111,112,113,114,115,116,117,118,119,120,121]. Using plants as sources of fibrinolytic substances seems to be an attractive prospect for future research, but as yet, most of the available data derive from basic in vitro screening tests employing whole blood or plasma fibrin clots (Table 5). Unfortunately, very few in vivo studies are devoted to pro-fibrinolytic action of plant extracts. However, in mice, administration of 200 mg/mL and 400 mg/kg of body weight doses of ethanolic extract of *Ocimum basilicum* L. shoots has been found to stimulate the fibrinolytic system, resulting in a higher content of fibrin degradation products (FDPs) [122]. In humans, supplementation with a dried garlic powder (900 mg/day; for 14 days; placebo-controlled study) increased the t-PA activity but did not influence PAI-1 or fibrinogen levels [123]. Favorable changes in plasma clotting, clot formation, and lysis were found in middle-aged patients with metabolic syndrome taking 100 mg of *Aronia melanocarpa* (Michx.) Elliott extract (three times a day, for 2 months) [124]. It is assumed that in simple experimental systems, the observed effects may be a result of the presence of plant proteases or modulation of the fibrinolytic enzymes by members of various classes of phytochemicals that are present in plant material. At the level of the whole organism, molecular mechanisms of pro-fibrinolytic or anti-thrombotic actions are much more complicated. The plant-derived substances are able to affect diverse components of the hemostatic system [125,126], and their final effects may be a result of stimulation of the fibrinolytic system, modulation of the coagulation cascade and platelet activity, or other activities.

However, in some cases, substances that the most likely display the fibrinolytic activity have been at least preliminarily identified. For instance, an edible and medicinal plant *Aster yomena* (Kitam.) Honda has been found to synthesize a protease (named kitamase) with thrombolytic (plasmin-like) and anticoagulant properties. Kitamase preferentially digested the Aα- and γ-γ chains of fibrin(ogen). It also acted as an anticoagulant, i.e., prolonged the coagulation time, the activated partial thromboplastin time, and prothrombin time [127]. Experiments on thrombolytic potential of *Gastrodia elata* Blume demonstrated that ethyl acetate and water extracts enhanced plasmin activity. More in-depth analyses revealed three the most active compounds from these extracts, i.e., 4-hydroxybenzyl alcohol, 4-hydroxybenzaldehyde, and 4-ethoxymethylphenol, that significantly stimulated plasmin activity [128]. In studies on *Lagenaria siceraria* (Molina) Standley fruit extract, kaempferol was found to be a key ingredient of this plant preparation, responsible for stimulating fibrinolytic activity in blood clots [129]. However, in other studies, kaempferol was found to inhibit serine proteases and impair the activity of complement proteins [91].

Thrombolytic activity was also demonstrated for plant latex proteases. Interestingly, most of the identified plant latex proteases display both thrombin-like (pro-coagulant) and plasmin-like activity. The thrombin-like activity of plant proteases has been found in in vitro and in vivo investigations. Plant latex proteases can hydrolyze (but with different efficiency) all fibrinogen subunits (i.e., Aα, Bβ, and γ chains) leading to the clot degradation; however, they can also stimulate fibrin formation by partial degradation of fibrinogen, involving removal of fibrinopeptides FpA and FpB from N-terminal regions of the fibrinogen Aα and Bβ chains, respectively [130,131]. During the blood coagulation cascade, hydrolytic release of these peptides from fibrinogen by thrombin is critical for starting its polymerization and formation of the fibrin network. Comparative analyses of hydrolytic activities of *Calotropis gigantea* R. Br., *Synadenium grantii* Hook. f., and *Wrightia tinctoria* R. Br. latex extracts demonstrated that Aα chains of fibrin(ogen) are the most susceptible to degradation by the examined latex proteases, followed by Bβ and γ chains. The clot dissolving properties were as follows: *C. gigantea* > *S. grantii* > *W. tinctoria*. The same pattern of efficiency of the examined extracts was observed during the fibrin clot formation tests, after recalcification of blood plasma [132]. Pergularain e I, a cysteine protease (23.3 kDa, N-terminal sequence: LPHDVE) from *Pergularia extensa* (Jacq.) N.E.Br. latex displayed both thrombin-like and thrombolytic properties [133]. Hirtin, a 34 kDa serine protease with the N-terminal sequence YAVYIGLILETAA/NNE was identified in the latex of a medicinal herb *Euphorbia hirta* L. The enzyme had a broad range of hydrolytic activity, including azocaseinolytic, gelatinolytic, fibrinogenolytic, and fibrinolytic properties (with the following preference: Aα > Bβ > γ units) [134]. Another serine protease (AMP48) displaying plasmin-like activity was isolated from *Artocarpus heterophyllus* Lam. latex. A 48-kDa enzyme, characterized by the N-terminal aa sequence AQEGGKDDDGG, was able to effectively hydrolyze Aα chains and partly degrade Bβ and γ subunits of human fibrinogen [135].

## 6. Concluding Remarks and Perspectives

Diverse classes of plant-derived substances have been evidenced to inhibit fibrinolytic proteins or to possess fibrinolytic activity. Both (poly)phenols and other low molecular secondary metabolites may be considered as promising templates for fibrinolysis inhibitors or modulators; however, given the ample evidence, the peptide-type substances displayed higher inhibitory efficiency. The Laskowski folds may be used for designing new structures or combinations with other substances to obtain plasmin inhibitors that could be useful in different diseases. However, low specificity of most of naturally occurring substances limits the possibility of undertaking studies in animals and is still a significant obstacle to developing effective fibrinolysis-targeting drugs.

SFTI-1 may be a template for different modifications and a base for development of new formulae of delivery of this potential drug. Both the STFI-1 structural scaffold and other scaffolds that are present in different plant-derived peptide-type inhibitors may be modified to obtain structures (e.g., by optimization of the sequence in the canonical loop), providing both increased inhibitory efficiency and selectivity. Some attempts to modify the Kunitz-type inhibitors have been also undertaken. The genetic engineering techniques enable modification of natural substances and their production systems. For example, a recombinant kallikrein inhibitor (rBbKI) and a recombinant cruzipain inhibitor from *B. bauhinioides* (rBbCI) were obtained by heterologous expression and production in the form of functional proteins in *Escherichia coli* [136]. Native BbCI and BbKI share over 80% sequence similarity; however, some important divergences have been found in their reactive sites. This was the context for the development of recombinant BbKI with a single mutation replacing the Arg64 residue with Ala. The R64A mutation led to significant modification of BbKI specificity to a weaker inhibitor of plasma kallikrein than the wild-type rBbKI (K_i_ changed from 2 to 98 nM) and a strong inhibitory action towards plasmin (K_i_ changed from 33 to 2.6 nM) [137].

Furthermore, the latest interesting trends in research on fibrinolysis modulators include other ways of exploiting the potential of plant-derived substances, e.g., the use of modified nanoparticles (NPs) as drug carriers. Recently, Yu and co-authors [138] described the polyphenol-based nanocarriers of thrombolytic drugs. The nanoparticle concept was used to improve drug delivery system using TA, which is capable of interacting with diverse surfaces and target molecules through a wide spectrum of mechanisms, including hydrophobic, hydrogen bond, covalent bond, and electrostatic interactions; metal coordination; and π interactions. TA was used to cross-link u-PA-loaded sacrificial mesoporous silica template and a thrombin-cleavable peptide to obtain the bioresponsive NP carrier of u-PA. Preliminary in vitro assessments of the nanocarrier action demonstrated an increase in Plg activation in the presence of thrombin (1.14-fold) and lower association with macrophages and monocytes. Further, an animal study revealed delayed blood clearance of the modified nanocarriers (90% clearance at 60 min) compared to the mesoporous silica template NPs (90% clearance at 5 min).

Therefore, the latest approach based on the use of natural pharmacophore templates and on investigation of their modifications could offer a promising way to overcome the difficulties related to low specificity of natural compounds.

## Figures and Tables

**Table 1:** General characteristics of main proteins of the fibrinolytic system.

Protein of the Fibrinolytic System	Function	Molecular Weight (Da)/Number of Amino Acids	Catalytic triad of Enzymes/Reactive Site of Inhibitors	Main Origins	Plasma Concentration: µg/mL (Molar)	Plasma Half-Life (T_1/2_)
Plasminogen	proenzyme of plasmin	92,000/791	-	Liver	180 (2 µM)	~2.2 days
Plasmin (EC 3.4.21.7)	serine protease	85,000/530	His603, Asp646, Ser741	Liver	180 (2 µM)	
t-PA (EC 3.4.21.68)	serine protease (plasminogen activator)	70,000/527	His322, Asp371, Ser478	endothelial cells	0.005 (70 pM)	~5–6 min
u-PA (EC 3.4. 21.73)	serine protease (plasminogen activator)	53,000/411	His204, Asp255, Ser356	kidney, lung, and many other cell types	0.002 (40 pM)	~8–10 min
uPAR	urokinase receptor	55,000/313	-	various cell types	-	
α_2_-antiplasmin	serine protease inhibitor (SERPIN)	70,000/464	Arg376-Met377	Liver	70 (1 µM)	~2.6 days
α_2_-macroglobulin	serine protease and metalloproteinase inhibitor	725,000/5804 (tetramer)	the “*bait region*” and the reactive site formed by thiol esterification of β-SH group of Cys-949 and γ-carbonyl group of Glx-952	Liver	2000–4000 (2.4–4.8 µM)	5–8 days
PAI-1	serine protease inhibitor (SERPIN)	52,000/379	Arg346-Met347	liver, endothelial cells, megakaryocytes, smooth muscle	<0.05 (<1 nM)	1–2 h
PAI-2	serine protease inhibitor (SERPIN)	47,000 (non-glycosylated form) or 60,000 (glycosylated form)/393	Arg358-Thr359	placenta, monocytes/macrophages	<0.250 (during pregnancy)	24 h
TAFI/TAFIa	procarboxypeptidase B/carboxypeptidase B—fibrinolysis inhibitor	60,000/401		Liver	4–15 (73–275 nM)	~10 min (TAFIa)

**Table 2:** Currently used anti-fibrinolytic drugs.

Molecular Action	Name	Biochemical Mechanisms of Action	Pharmacokinetics/ Elimination Route	Exemplary Clinical Use	Side Effects
Inhibition of plasminogen activation to plasmin	Epsilon aminocaproic acid (EACA; 6-aminohexanoic acid)	synthetic lysine analogues; competitive inhibitors of plasminogen binding to fibrin (and its activation to plasmin)	terminal elimination T_1/2_ of 2 h/renal	Cardiac, prostate, gynecological and other surgery; bleeding associated with hyperfibrinolysis; bleeding associated with hematopoietic disorders	Hypotension, nasal and conjunctival congestion, gastrointestinal disturbances (diarrhea, nausea, vomiting, abdominal pain), dizziness, headache, tinnitus, and ejaculation disorders [31]
	Tranexamic Acid (TXA; *trans*-4-(aminomethyl)cyclohexanecarboxylic acid)		terminal elimination T_1/2_ of 2–3 h/renal	Cardiac, orthopedic, gastrointestinal, and other surgery [32]	Hypotension; longer-term administration may induce rashes, nausea, vomiting, weakness, retrograde ejaculation, myopathy, and rhabdomyolysis [32]
Inhibition of plasmin	Aprotinin	polypeptide inhibitor of serine proteinases, isolated from bovine lung, forms reversible complexes with the active serine residue in proteases and inhibits not only plasmin but also kallikrein, chymotrypsin, and trypsin	terminal elimination T_1/2_ of 10 h/mainly by proteolysis, <10% renal	Cardiac surgery	Renal failure, myocardial infarction, heart failure, stroke, encephalopathy, mortality [33], anaphylaxis, graft occlusion, and stroke [34]

**Table 3:** The plant-derived substances with the highest potency in vitro to inhibit the serine proteases. Compounds with IC_50_ ≥ 50 µM have been considered weak inhibitors, and therefore they are not listed in this table. K_d_—dissociation constants of the serine protease-plant-derived compound complexes; IC_50_—half-maximal inhibitory concentration.

Compound	Inhibited Enzyme	K_d_	IC_50_	References
Bufadienolides	Plasmin		16.70 μg/mL	[43]
	Thrombin		2.79 µg/mL	[44]
Cyanidin	Thrombin		0.25 μM	[45]
	Factor Xa		3.0 μM	[46]
Epigallocatechin gallate (EGCG)	Elastase		0.4 μM	[47]
Elagic acid	Thrombin	7.6 µM		[48]
Esculin	Urokinase		25.0 µM	[49]
Hypericin	Trypsin		4.5 μM	[50]
	Urokinase		30.7 μM	
Hyperoside	Elastase		0.3 μM	[51]
	Trypsin		14.5 μM	[50]
	Urokinase		8.3 μM	
Myricetin	Thrombin		6.0 μM	[52]
	Elastase		4.0 μM	[47]
Procyanidin B2	Factor Xa		1.2 µM	[46]
Rutin	Plasmin	10.7 µM		[53]
	Thrombin		25.0 µM	[49]
	Trypsin		40 µM	
	Urokinase		6.0 µM	
Salicin	Thrombin		11.4 µM	[50]
Sennoside A	Trypsin		6.1 µM	[50]
Sennoside B	Trypsin		10.6 µM	[50]
Silybin	Thrombin		20.9 µM	[50]
			25 µM	[45]
	Trypsin		3.7 µM	[50]
	Urokinase		21.0 µM	
	Factor Xa		35.0 µM	[46]
Quercetin	Plasmin	0.62 µM		[53]
	Thrombin	0.35 µM		[48]
			30.0 µM	[50]
			1.5 µM	[45]
			35.0 µM	[49]
	Factor Xa		5.5 µM	[46]
	Trypsin		15.4 µM	[50]
			10.0 µM	[49]
	Urokinase		12.1 µM	[50]
			20.0 µM	[49]
	Elastase		50.0 µM	[50]
Quercetin-3-glucuronide	Plasmin	0.14 µM		[54]
5,5-trans-fused cyclic lactone euphane triterpene (GR133487)	Trypsin		0.12 µM	[55]
	Chymotrypsin		0.01 µM	
	Thrombin		0.004 µM	
	Factor Xa		> 10 µM	
	Factor XIa		1.1 µM	
	Factor XIIa		> 10 µM	
	t-PA		> 10 µM	
	Plasmin		5.6 µM	
	Elastase		> 10 µM	
	Cathepsin G		> 10 µM	
5,5-trans-fused cyclic lactone euphane triterpene (GR133686)	Trypsin		0.07 µM	[55]
	Chymotrypsin		0.07 µM	
	Thrombin		0.04 µM	
	Factor Xa		> 10 µM	
	Factor XIa		0.7 µM	
	Factor XIIa		> 10 µM	
	t-PA		1.0 µM	
	Plasmin		3.5 µM	
	Elastase		> 10 µM	
	Cathepsin G		2.2 µM	

**Table 4:** Peptide-based plant inhibitors of serine proteases of the blood coagulation cascade and fibrinolytic system.

Type of Inhibitor	Name	Plant Source	Target Serine Protease	K_i_	References
Bowman-Birk	TcTI	*Torresea cearensis* Allemão	Plasmin	36.0 nM	[57]
			Factor XIIa	1.45 µM	
Kunitz	ApTIA	*Acacia plumosa* Lowe	Kallikrein	0.55 µM	[58]
	ApTIB			0.58 µM	
	ApTIC			0.65 µM	
	AaTI	*Araucaria angustifolia* (Bertol.) Kuntze	Plasmin	7.0 µM	[59]
	BbKI	*Bauhinia bauhinioides* (Mart.) J.F.Macbr.	Kallikrein	0.35 nM	[60]
			Plasmin	33.0 nM	[61]
			Factor XIIa	110.0 nM	
	BuXI		Plasmin	76.0 nM	
			Factor Xa	14.0 nM	
			Factor XIIa	74.0 nM	
	BvTI		Kallikrein	23.0 nM	
			Factor XIIa	21.0 nM	
	BrTI		Kallikrein	14.0 nM	
	CeKI	*Caesalpinia echinata* Lam.	Plasmin	0.18 nM	[62]
			Factor XIIa	0.18 nM	
			Factor Xa	490 nM	
			Kallikrein	3.1 nM	
	Corn Hageman factor inhibitor	*Zea mays* L.	Factor XIIa	2.1 nM	[63]
	STI	*Glycine max* (L.) Merr.	Plasmin	192.10 nM	[64]
			Factor XIIa	1367.59 nM	
			Kallikrein	5.70 nM	
	SWTI	*Swartzia pickelii* Killip	Plasmin	2.52 nM	[65]
			Kallikrein	20.25 nM	
	Tamarind Kunitz inhibitor	*Tamarindus indica* L.	Factor Xa	220.0 nM	[66]
Potato-type PI	CMTI-V	*Cucurbita maxima* Lam.	Factor XIIa	41.0 nM	[67]
Squash PI	MCTI-I	*Momordica charantia* L.	Factor XIIa	13 nM	[68]
			Kallikrein	110 μM	
			Factor Xa	100 μM	
	MCTI-II	*Momordica charantia* L.	Factor XIIa	56 nM	
			Kallikrein	100 μM	
			Factor Xa	1.4 μM	
			Factor XIa	18 μM	
	MCTI-III	*Momordica charantia* L.	Factor XIIa	1.6 μM	
			Kallikrein	140 μM	
			Factor Xa	59 μM	
	CMTI-III	*Cucurbita maxima* Lam.	Factor XIIa	70 nM	
			Kallikrein	130 μM	
			Factor Xa	23 μM	
	LLTI-II	*Lagenaria leucantha* Rusby var. *Gourda Makino*	Factor XIIa	1.4 μM	
			Kallikrein	27 μM	
			Factor Xa	41 μM	
	LLTI-III	*Lagenaria leucantha* Rusby var. *Gourda Makino*	Factor XIIa	4.2 μM	
			Kallikrein	200 μM	
			Factor Xa	19 μM	
	LCTI-II	*Luffa cylindrica* Roem.	Factor XIIa	75 nM	
			Kallikrein	20 μM	
			Factor Xa	780μM	
	LCTI-III	*Luffa cylindrica* Roem.	Factor XIIa	3.8 nM	
			Kallikrein	38 μM	
			Factor Xa	100 μM	

**Table 5:** Exemplary data from in vitro studies on effects of plant-derived preparations on fibrin clot lysis. SK—streptokinase.

The Examined Plants	Biological Materials and the Used Concentrations of Plant Extracts	Reference Fibrinolytic Drug	Main Findings	References
*Allium affine* Ledeb.	Human blood clots; hydro-alcoholic extract from aerial parts of *A. affine* (70% ethanol); 0.005–50 mg/mL	SK (8000 IU)	*A. affine* efficiency—up to ~30% of clot lysis; SK—51.40%	[103]
*Allium elburzense* Wendelbo	Human blood clots; hydroalcoholic (70% ethanol), aqueous, chloroformic and butanolic extracts from *A. elburzense* bulb; 5 mg/mL	SK (30,000 IU)	Clot lysis efficiency: aqueous extract (33.11%) > hydroalcoholic extract (22.40%) > butanolic extract (16.75) > chloroformic (9.77%) extract; SK—60.59%	[104]
*Anona senegalensis* (A.DC.) Pichon, *Buchholzia coriacea* Engl., *Citrullus colocynthis* (L.) Schrad., *Cnidoscolus aconitifolius* (Mill.) I. M. Johnst., *Curculigo pilosa* (Schumach. & Thonn.) Engl., *Nicotiana tabacum* L., *Parinari curatellifolia* Planch. ex Benth., *Peperomia pellucida* (L.) Kunth, *Sida acuta* Burm. f., *Xylopia aethiopica* (Dunal) A.Rich.	Human blood clots; the crude methanolic extracts; 100 μg/mL	SK (30,000 IU)	The highest clot lysis efficiency: *P. curatellifolia*—56.12%, *C. aconitifolius* 48.38%, *A. senegalensis*—46.36%, *X. aethiopica* —43.20%, *B. coriacea*—27.06%; SK—60.20%	[105]
*Byttneria pilosa* Roxb.	Human blood clots; crude, methanol extract of *B. pilosa*; 100 µL/clot sample	SK (30,000 IU)	Clot lysis in samples treated with *B. pilosa* 46.20 ± 2.27%; in samples treated with SK: 82.60 ± 2.45%	[106]
*Campomanesia xanthocarpa* (Mart.) O. Berg	Human and mice blood clots; water extract from *C. xanthocarpa* leaves; 1, 3, 10, 30, and 100 μg/mL	SK (1, 3, 10, 30, and 100 μg/mL)	In mice blood, EC_50_ for *C. xanthocarpa* extract and SK: 21 μg/mL and 24 μg/mL, respectively; the E_max_ (at 100 μg/mL) was 56 ± 9% and 60 ± 14%, respectively. In human blood, the EC_50_ for *C. xanthocarpa* extract and SK was 11 and 4 μg/mL, respectively; E_max_ = 62 ± 7% and 70 ± 6%, respectively	[107]
*Cnidoscolus aconitifolius* (Mill.) I.M. Johnst.	Human blood clots; aqueous (Aq), ethanolic (EtOH), acetonic (An), ethyl acetate (AcOEt), diethyl ether (Et_2_O), and hexane (Hx) extracts from *C. aconitifolius* leaves; 0.1, 1, and 10 mg/mL	SK (30,000 IU)	The most effective were AcOEt, An, and Hx extracts (at 10 mg/mL), yielding 18.4—24% lysis; SK—76% lysis	[108]
*Fagonia arabica* L., *Saussurea lappa* Decne., *Tinospora cordifolia* Thunb.	Human blood clots; aqueous extracts from whole dry plant of *F. arabica*, dry bark of *S. lappa*, dry stem of *T. cordifolia*; 100 µL/clot sample	SK (30,000 IU)	Clot lysis efficiency: 68.06%, 14.85%, 25.01%, and 92.54%, for *F. arabica*, *S. lappa*, *T. cordifolia*, and SK, respectively.	[109]
*Commelina benghalensis* Forssk.	Human blood clots; methanol extract from *C. benghalensis* leaves; 10–1000 μg/mL	SK (30,000 IU)	Average lytic activity of 40.94% for the extract, and 75% for SK	[110]
*Cucumis dipsaceus* Wender. ex Steud.	Fibrinogen preparation; 25 and 50 µg/mL	-	Fibrinogenolytic activity of the aqueous *C. dipsaceus* dialysate fraction (AqCDF): hydrolysis of Aα and Bβ chains of fibrinogen, partial degradation of γ chain	[111]
*Curculigo recurvata* W.T. Aiton (Satipata)*, Amorphophallus bulbifer* Roxb. (Olkachu), *Phyllanthus sikkimensis* Muell. Arg., *Thunbergia grandiflora* Roxb. (Nillata)	Human blood clots; methanol extracts from the examined plants; 100 µL/clot sample	SK (30,000 IU)	Clot lysis efficiency: *C. recurvata* 28.10%, *A. bulbifer* 42.47%, *P. sikkimensis*—32.86%, *T. grandiflora* 25.51%, SK—78.23%	[112]
*Litchi chinensis* Sonn.	Rat fibrin clots; ethanol (70%) extract from dried fruits; fibrin plate assay, 0.5, 1, 2, and 4 mg/mL	Plasmin, 1–2 µg/fibrin clot plate	Induction of fibrinolysis at 2 and 4 mg/mL	[113]
*Maba buxifolia* (Rottb.) Juss.	Human blood clots; methanol extract from the plant stem; 1 mg/mL	Urokinase (50,000 IU)	24.3% of clot lysis for the extract; 100% lysis for urokinase-treated samples	[114]
*Mentha arvensis* L., *Mentha spicata* L., *Mentha viridis* L.	Human blood clots; *Mentha* sp. extracts (10 mg/mL): 95% methanol, 99.50% ethanol, 95% chloroform and acetone extracts); 100 µL/clot sample	SK (15,000 IU)	32.56%, 30.89%, 30.29% clot lysis for the methanol extracts; 32.04%, 30.37%, 30.02% clot lysis for the ethanol extracts; 31.87%, 29.77%, 29.05% clot lysis efficiency for the chloroform extracts; and 30.29%, 28.45%, 27.55% clot lysis efficiency for the acetone extracts for *M. arvensis*, *M. spicata*, and *M. viridis*, respectively	[115]
*Ocimum tenuiflorum* Burm.f., *Andrographis paniculate* (Burm.f.) Wall., *Adhatoda vasica* Nees, *Leea macrophylla* Roxb., *Litsea glutinosa* (Lour.) C.B.Rob.	Human blood clot; methanol extracts; 100 µL/clot sample	SK (30,000 IU)	*L. macrophylla* extract had the highest activity (47.47% of clot lysis); SK—71.14% of clot lysis	[116]
*Tinospora cordifolia* (Willd.) Miers, *Rubia cordifolia* L., *Hemidesmus indicus* (L.) R. Br. ex Schult., *Glycyrrhiza glabra* L., *Fagonia arabica* L., *Bacopa monnieri* Hayata & Matsum.	Human blood clots; aqueous herbal extracts; 100 µL/clot sample	SK (30,000 IU)	*F. Arabica*—75.6% clot lysis, *B. monnieri*—41.8% clot lysis, other extracts < 20% clot lysis; SK—86% clot lysis	[117]
*Trema orientalis* L., *Bacopa monnieri* Hayata & Matsum., *Capsicum frutescens* L., *Brassica oleracea* L., *Urena sinuata* L.	Human blood clots; methanol crude extracts and fractions (i.e., chloroform, n-hexane, hydro-methanol, and ethyl acetate fractions); 100 µL/clot sample	SK (30,000 IU)	The highest clot lysis activity: chloroform fractions from *T. orientalis*, *B. monnieri*, *C. frutescens*, *B. oleracea*, and *U. sinuata*, i.e., 46.44 ± 2.44%, 48.39 ± 10.12%, 36.87 ± 1.27%, 30.24 ± 0.95%, and 47.89 ± 6.83%, respectively; SK: 80.77 ± 1.12%	[118]

## Data Availability

Not applicable.
